# Strategic applications of heterocyclic compounds in pharmaceutical innovation: a business perspective

**DOI:** 10.3389/fchem.2026.1780357

**Published:** 2026-04-28

**Authors:** Yan Dong, Jindong Dai, Yuerong Zou, Kanagaraj Rajalakshmi, Natarajan Kiruthiga, P. Balaji, Dongwei Zhu

**Affiliations:** 1 Department of Immunology and Laboratory Medicine, Jiangsu Key Laboratory of Laboratory Medicine, School of Medicine, Jiangsu University, Zhenjiang, China; 2 School of Chemistry and Chemical Engineering, Jiangsu University, Zhenjiang, China; 3 Department of Pharmaceutical Chemistry, KMCH College of Pharmacy, Coimbatore, Tamil Nadu, India; 4 Department of Pharmaceutical Chemistry and Analysis, School of Pharmaceutical Sciences, Vels Institute of Science, Technology and Advanced Studies, Chennai, Tamil Nadu, India

**Keywords:** business strategy, drug development, heterocyclic chemistry, market analysis, medicinal chemistry, pharmaceutical innovation, sustainability

## Abstract

The heterocyclic compound is one of the finest spanned backbones for developing most innovative agents in the pharmaceutical industry. These adaptable molecules have played an important role in the discovery of therapeutic agents of a wide range, including antibiotics, anticancer drugs and antivirals. The role of heterocyclic chemistry in pharmaceutical innovation is thus one that extends beyond mere structural complexity but contributes significantly to improved therapeutic index, and in doing so stimulates economic activity in the field. The medicinal significance of heterocycles, their role in drug discovery, and their commercial prospects are discussed in the Research article. It explores types of heterocyclic compounds, its historical importance, influence on the world health and market trends delivering its industrial usage. Synthetic challenges, regulatory hurdles, and sustainability concerns are discussed in tandem with future opportunities that may arise from advancements in green synthesis and artificial intelligence (AI)-driven drug discovery. This study emphasizes the transformative potential of heterocyclic compounds in modern medicine by connecting their scientific advancements to business strategies.

## 1 Introduction

**FIGURE 1:**
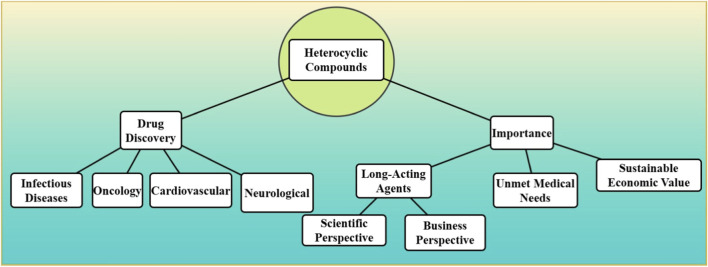
The strategic role of heterocyclic compounds in drug discovery, highlighting their therapeutic applications in infectious diseases, oncology, cardiovascular, and neurological treatments, and their business and scientific significance.

One such class of compounds, heterocyclic compounds, which contain at least one other atom in their ring structure aside from carbon, make up the molecular foundation of many of today’s most common and widely used drugs (Bansal, 2020; Omar, 2020). These compounds have evolved over the decades to become extremely versatile, successful agents in targeting different biological pathways, forming the backbone of pharmaceutical innovation. Heterocyclic compounds altered drug discovery, from their use in treating infectious diseases to their successful contribution toward oncology, cardiovascular, and neurological therapeutics. Their role in treating critical human suffering ultimately, it is crucial to know why and how long-acting agents are so important (both scientifically and from a business perspective) to make the best use of them and realize their full potential to address serious unmet medical needs while also generating sustainable economic value for the pharmaceutical industry Figure 1.

Heterocycles are structurally defined as chemical compounds with a ring in which at least one atom is not carbon. The structural diversity of these compounds also permits the design of highly specific molecules capable of interacting with a wide variety of biological targets from enzymes and receptors to ion channels and DNA. That versatility has resulted in some of the most successful drugs in history. Several drugs, such as Imatinib (Gleevec) — a tyrosine kinase inhibitor used in the treatment of chronic myelogenous leukemia — and Zidovudine (AZT) — an antiviral used in the treatment of HIV — are also heterocycles. As diseases become ever more complex and the need for precision medicine ever greater, so the demand for new heterocyclic-based drug candidates, which provide specific therapeutic effects with low side effects, has grown (Zhang et al., 2020; Zhang et al., 2022).

The drug development of heterocyclic compounds has great significance in business. These are some of the reasons why pharmaceutical companies are paying more attention to these compounds given their effectiveness, ability to potentially be cheaper than large molecule medicines, and wide applicability in multiple therapeutic areas. The specialisation and growth in research of heterocyclic drugs has led to considerable investment being made in drug R&D, along with strategic collaborations among leading pharmaceutical companies, academic institutions to research chemical compounds and biotechnology organisations. The pharmaceutical industry, valued at more than a trillion dollars, is experiencing a dramatic paradigm shift, with heterocyclic compounds at the forefront of the new wave of pharmaceuticals and the progress that will pave the way for the future (Amin et al., 2022; Kumar and Goel, 2022).

Therefore, the business significance of heterocyclic compounds is extensive encompassing market aspects, intellectual property, regulatory aspects, and commercialization in pharmaceutical companies. These factors must be cautiously evaluated by companies to develop drug-based marketing strategies for heterocyclic compounds. The task they must consider such as Intellectual property (IP) protection which is one of the most accessible one so essentially the patenting of new heterocyclic drug candidates has the potential to be strategically important for a company’s competitive positioning in the marketplace. In addition, the time taken for the drug to transition from discovery to sales is a critical factor in its potential results, and heterocyclic compounds typically have a faster progression due to better known chemistry and favorable pharmacokinetic properties. There is a myriad of successful methods however one of the most important currently in pharmaceuticals is the evolution of personalized medicine; a drug tailored to a person’s genes profile. This emerging trend is highly suitable for their designing since heterocyclic compounds can be engineered with high specificity. Such compounds used in precision medicine requires knowledge of genetic markers and biological pathways that are specific to certain conditions, enabling the creation of drugs targeting the actual causes of diseases instead of treating symptoms. With heterocyclic compounds being at the forefront of this revolution (de La Torre and Albericio, 2021; Kapoor et al., 2025; Reddy et al., 2022).

Historically, natural products have been an invaluable source of drug discovery that offers structurally diverse heterocyclic scaffolds that have proven bioactivity (Ou et al., 2025). Naturally occurring heterocyclic systems are the inspiration of many clinically successful heterocyclic drugs, such as Morphine, Quinine, Camptothecin, and Vincristine (Chen et al., 2025; Theivendren et al., 2025). These compounds that have presented significant clues towards the synthesis of synthetic analogs that have better pharmacokinetic and pharmacodynamic properties (Li et al., 2024; Ramesh et al., 2023). Further efforts to research natural product chemistry especially in the field of ethnopharmacology and biodiversity prospecting are still a corner stone in the discovery of new heterocyclic motifs to use in pharmaceutical practice (Theivendren et al., 2022). Complementarily, the principles of green chemistry have revolutionized the methods of synthesis that are used in the production of heterocyclic drugs. The atom-economic processes, solvent-free conditions and recyclable catalysts not only lessen the burden on the environment but also enhance the efficiency and scale of the process. A pharmaceutical manufacturing process is now guided by green chemistry, and through it, cost-effective and sustainable production of heterocyclic therapeutics is possible.

**Figure:**
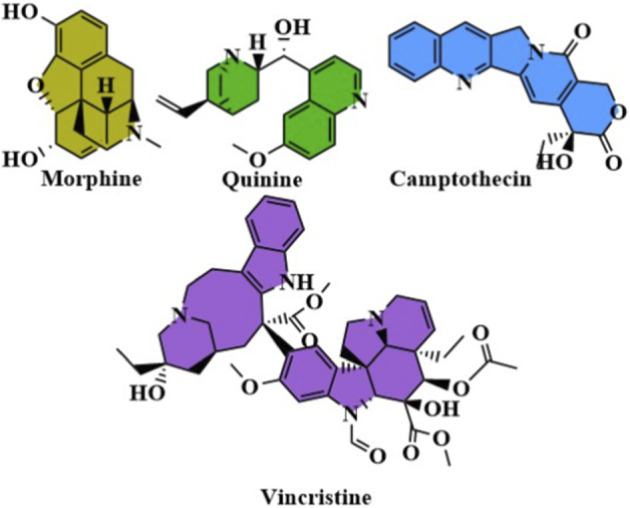


Besides precision medicine, also the business aspect of heterocyclic compounds correlates with cost-efficiency. As healthcare costs are rising across the globe, pharmaceutical companies are demanding researchers to minimize the cost of drug development while ensuring a well-established efficacy and safety profile. Due to the most pre-established synthetic routes of heterocyclic compounds, they often pose lower costs of development and production. The reworking to new indications of existing heterocyclic drugs has also become an attractive business approach. This allows companies to capitalize on the safety profiles and knowledge of heterocyclic compounds they already have, while also entering new revenue streams in therapeutic areas that have yet to be significantly developed (Pollok and Waldvogel, 2020).

**FIGURE 2:**
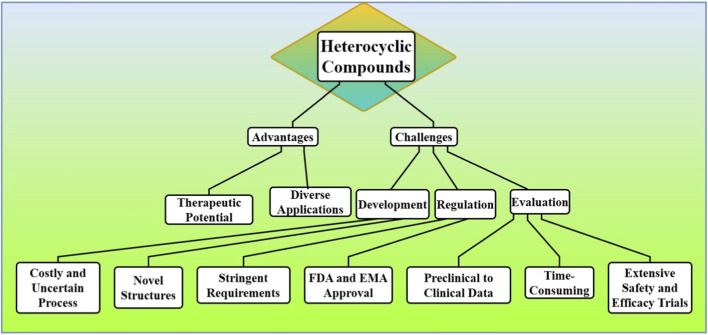
The challenges in developing and commercializing heterocyclic compounds, focusing on costly and uncertain drug development processes, stringent regulations, and the extensive data required for FDA and EMA approval.

Notwithstanding the excessive advantages of heterocyclic compounds, their development and commercialization face reopening challenges in the pharmaceutical enterprise. Another major issue is the regulation, which is getting increasingly stringent. One of the (many) challenges in drug development involves the long, costly, uncertain process for evaluating new drugs, especially those based on novel heterocyclic structures. Agencies like the U.S. Food and Drug Administration (FDA) or the European Medicines Agency (EMA) require cross the board preclinics to clinics data, to prove the efficacy and safety of drugs, which can extensive the time of market Figure 2. Moreover, the ever-increasing threat posed by generic drugs and the rising demand for rapid, economical treatment options make the commercial landscape rather treacherous for heterocyclic based drugs.

It is a challenge related to the increasing epidemic of drug resistance, especially in the case of antibiotics and antiviral agents. Many pathogens have over time developed resistance to conventional drug therapies, diminishing drug efficacy. This phenomenon underscores the necessity for continued discovery of new heterocyclic structures with novel modes of action. For pharmaceutical companies, however, this is both a challenge (and an opportunity) to capture the market share developing the next-generation antibiotics and antivirals (Han et al., 2025; Pollok and Waldvogel, 2020; Wang et al., 2021).

Heterocyclic compounds open up new avenues for the discovery and development of therapeutic agents, and challenges involved with their use is presently being heavily researched. The field of computational chemistry, artificial intelligence, and high-throughput screening methods have all progressed significantly, which has allowed for the quick identification and optimization of novel heterocyclic candidates. This should help to accelerate drug discovery, shorten development times, and lower costs, making heterocyclic-based drugs even more commercially viable. In addition, the growing trend towards global health challenges, including cancer, diabetes, and infectious diseases, signifies a promising market for heterocyclic compounds capable of combating these ailments (Kopf et al., 2022; Verma et al., 2024; Wang et al., 2021).

Finally, the strategic diversity of heterocyclic functionality is a critical tool in medicinal development. This makes them increasingly applicable to drug development, which is in great demand for such therapeutic opportunities and approaches to critical health tournaments. Heterocyclic compounds hold considerable promise in trashing the pharmaceutical front from a business standpoint. Equally well, the crossroads of scientific discovery and commercial alternative drive the ideas of pharmaceutical enterprises in the light of the outstanding propagation of heterocyclic materials; specially groups such heterocycles are critical to not only nonsuch adjuvant to the necessary advancement of healing delivery systems, such as extenuators pharmacological openings in social pharmaceutical sets, but also the general illumination of commercial approaches and also turmoil missions (Biswas et al., 2024; Kopf et al., 2022; Sánchez et al., 2023; Wang et al., 2021).

## 2 Heterocyclic compounds in drug development

### 2.1 Overview of heterocyclic structures and medicinal properties

Heterocyclic compounds are organic compounds that contain rings containing at least one atom other than carbon (usually nitrogen, oxygen or sulfur). They are highly diversified scaffolds and can be classified into several types according to the number and nature of heteroatoms. Examples of these nitrogen heterocycles include pyridine, imidazole, quinoline and thiazole, which are prominent in medicinal chemistry for their individual chemical and biological properties. Heterocyclic compounds possess unique electronic features due to the presence of heteroatoms, allowing them to interact with biological targets in manners frequently unavailable to compounds with carbon-only architectures (Kabir and Uzzaman, 2022; Qadir et al., 2022). Heterocycles show a broad spectrum of medicinal applications; thereby, heterocycles are also one of the major components in gaining therapeutic agents to treat different diseases. For instance, many nitrogen-containing heterocyclics have been reported with antimicrobial, antiviral, and anticancer potential. Further, hetero-cyclic compounds including benzodiazepines and purine analogs play important therapeutic roles for neurological disorders and cancers, respectively. Heterocyclic compounds are essential for drug discovery because of their ability to form stable complexes with biological macromolecules, including proteins, enzymes, and nucleic acids. In fact, this has resulted in decades of the development of many life-saving drugs that are the central components of modern medicines. Multi-Panel representation of 2D chemical structure of: (a) Pyridine, Pyrimidine, Purine (nitrogen heterocycles); (b) Thiophene, Thiazole (sulfur heterocycles); (c) Furan, Oxazole (oxygen heterocycles); (d) Representative drugs: Imatinib (pyridine), Ciprofloxacin (quinolone), Metronidazole (imidazole), Sildenafil (pyrazolopyridinone), Oseltamivir Representation of heterocyclic scaffolds and approved drugs with heterocyclic structures have incorporated heterocyclic frameworks, which show the structural range and pharmacological significance of heterocyclic nitrogen, sulfur, and oxygen ring systems (Ali et al., 2022; Li et al., 2025).

**Figure:**
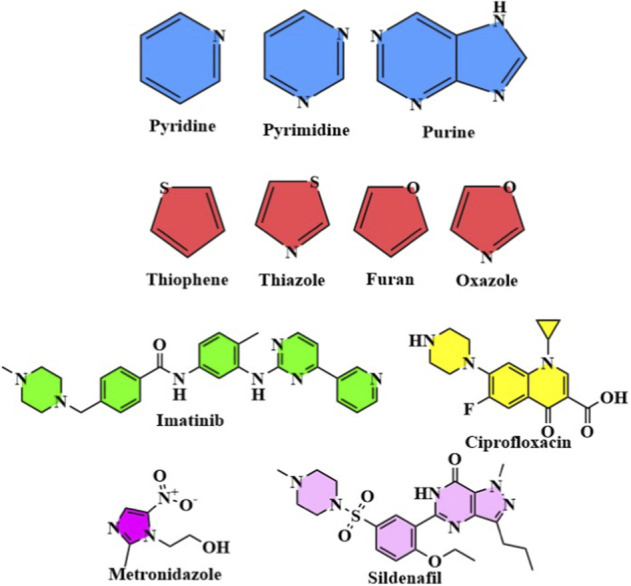


### 2.2 Historical milestones in heterocyclic chemistry

Heterocyclic chemistry dates to the beginning of the 19th century, and major advancements were made that would eventually make heterocycles commonplace in medicinal chemistry. The first milestones were in 1826 when the German chemist August Wilhelm von Hofmann synthesized from coal tar the first nitrogen-containing heterocycle, pyridine. This discovery was a critical breakthrough, as it led to the study of the nitrogen-containing heterocyclic compounds and their wide-ranging chemical reactivity. It all started with the discovery of quinoline by Carol Dietrich back in the mid-1800s in 1843 which, finally, set the ball rolling for the great interest in medicinal properties of heterocycles, mostly towards the treatment of malaria. A heterocyclic compound, quinoline, has shown significant antimalarial properties, and various chloroquine derivatives were touted as an important step for pharmacophore development in the 20th century. A further key milestone was the early 20th-century synthesis of benzodiazepines, a class of heterocyclic compounds that transformed the treatment of anxiety and insomnia. Diazepam (Valium), one of the first drugs specifically developed as an anxiolytic, was synthesized in 1957 by Leo Sternbach, who demonstrated the therapeutic promises of heterocyclic compounds Table 1. The unprecedented rise in heterocyclic-inspired antibiotics, antivirals, and anticancer therapeutics in the second half of the 20th century validated their importance in contemporary drug discovery and confirmed the structural foundation of heterocyclic chemistry in medicinal chemistry (Amin et al., 2022; Baranwal et al., 2023). i) Quinine (quinoline derivative) in the market worldwide as the antimalarial medicine, annual peak revenue of USD 500 million; (ii) Penicillin (beta-lactam) spawned USD 15 billion antimicrobial market; (iii) Diazepam/Valium (benzodiazepine) one of the most prosperous drugs in the 1970s–1980s and generated USD 2.3 billion at peak; (iv) Chlorpromazine (phenothiazine).

**TABLE 1:** The key milestones in the development of heterocyclic chemistry, highlighting significant discoveries and their impact on medicinal chemistry, particularly in the development of antimalarial, anxiolytic, and therapeutic drugs.

S.No.	Aspect	Key insights	Implications
1	Early milestone in heterocyclic chemistry	First nitrogen-containing heterocycle, pyridine, synthesized in 1826	Marked the beginning of the study of heterocycles in medicinal chemistry
2	Quinoline discovery	Quinoline discovered by carol dietrich in 1843, showing antimalarial properties	Paved the way for antimalarial drug development
3	Antimalarial properties	Quinoline and derivatives like chloroquine used to treat malaria	Key discovery for the development of malaria treatments
4	Benzodiazepine synthesis	First synthesized in the early 20th century, revolutionizing anxiety treatment	Benzodiazepines like diazepam became pivotal in treating anxiety and insomnia
5	Diazepam (Valium)	Synthesized in 1957 by Leo Sternbach, a major breakthrough in anxiolytic drugs	Marked a milestone in heterocyclic chemistry for treating anxiety
6	Rise of heterocyclic antibiotics	Heterocyclic compounds led to antibiotics, antivirals, and anticancer drugs	Boosted drug discovery in the treatment of infections and cancer
7	Therapeutic impact	Heterocyclic chemistry transformed various therapeutic areas in the 20th century	Expanded the scope of drug treatments in modern medicine
8	Structural foundation	Heterocyclic compounds confirmed as foundational in drug discovery	Established heterocycles as essential for medicinal chemistry
9	Continued advancements	20th century saw continued advancements in heterocyclic-inspired therapeutics	Heterocyclic compounds remain crucial for developing modern drugs
10	Importance in modern drug discovery	Heterocyclic compounds are integral to contemporary drug development	Ensures their ongoing relevance in pharmaceutical innovation

### 2.3 Role of heterocycles in creating FDA-approved drugs

Since the development of FDA-approved drugs, heterocyclic compounds have been discovered as important drug scaffolds, playing a monumental role in both modern pharmacology and medicine. Due to their capability to create stable and bioactive ligands with a high target specificity with respect to biological targets, they have become an indispensable asset in drug discovery. Heterocyclic compounds have unique properties (chemical stability, high distribution of electron density, similarity with natural biomolecules) that make them interact well with enzymes, receptors and DNA, serving as ideal candidates for drug design. The heterocyclic structures form the backbone of many FDA-approved drugs, especially in the fields of oncology, infectious diseases, and neurology. An important class of building blocks in drug discovery are heterocycles, e.g. Imatinib (Gleevec) (a pioneering cancer drug for chronic myelogenous leukemia) is based on a pyrimidine-based heterocycle. Another prominent example is Zidovudine (AZT), a purine-based heterocycle that was one of the first efficacious chemotherapeutics for the treatment of HIV/AIDS. Heterocycles also have a critical role in the structure of antibiotics: two quinolone-based compounds, Ciprofloxacin and Levofloxacin, are both essential treatments of bacterial infections. Aside from that, benzodiazepines such as Diazepam (Valium) recently became the first-tier drugs for treating anxiety and seizure disorders Table 2. These techniques showcase how heterocyclic chemistry has revolutionized drug development, resulting in efficient and targeted medications that have transformed the global healthcare landscape. Continued heterocycle exploration is indispensable for the discovery of new FDA-approved drugs into diverse therapeutic areas for unmet medical requirements (Chebanov et al., 2023; Martins da Silva et al., 2024). Case Study 1-Imatinib (Gleevec): A tyrosine kinase BCR-ABL inhibitor which is a pyrimidine compound. Discovery to approval period: 1990-2001 (11 years). Cost of development: -USD 1.2 billion. Protection term: 20 years; brought in USD 43 billion in cumulative sales to Novartis. Patent life in United States: 2015; in the market through follow-on patents. Case Study 2 - Oseltamivir (Tamiflu): A carboxylate antiviral which is cyclohexene. Discovery timeline: 1992-1999. The highest sales globally were USD 3.6 billion in the course of H1N1 pandemic (2009). The IP protection of Roche consists of synthesis and compound patents. Case Study 3- Sildenafil (Viagra): An inhibitor of PDE 5 pyrazolo pyrimidinone. Luckily discovered as a part of angina trials (1989). Time to approval: 1998. Expires 2012 (United States); has produced USD 2.0 billion per year at its zenith. IP expenses: The total cost of registering a full global patent portfolio (PCT route) is about USD 50,000-150,000 per active compound; and the long-term maintenance of patent exclusivity in the top markets is likely to cost USD 500,000-1,000,000.

**TABLE 2:** The role of heterocyclic compounds in the development of FDA-approved drugs, highlighting their significance in oncology, infectious diseases, neurology, and antibiotic treatments, driving advancements in global healthcare.

S.No.	Aspect	Key insights	Implications
1	Heterocyclic compounds in drug discovery	Heterocycles are crucial in modern pharmacology, forming stable and bioactive ligands	Essential for creating drugs with high target specificity and bioactivity
2	Role in oncology	Heterocycles like imatinib (Gleevec) are key in cancer treatment	Revolutionized cancer treatment, particularly for chronic myelogenous leukemia
3	Role in infectious diseases	Zidovudine (AZT) is a purine-based heterocycle used in HIV/AIDS treatment	One of the first effective treatments for HIV/AIDS.
4	Role in neurology	Benzodiazepines, like diazepam (Valium), treat anxiety and seizure disorders	Key treatments for anxiety and seizure management
5	Chemical properties of heterocycles	Heterocycles interact well with enzymes, receptors, and DNA.	Ideal for drug design due to chemical stability and similarity with biomolecules
6	Heterocycles in antibiotics	Quinoline-based drugs like ciprofloxacin and Levofloxacin treat bacterial infections	Essential for combating bacterial infections
7	Drug design and target specificity	Heterocyclic structures allow for targeted drug design	Enable high target specificity, improving drug efficacy
8	Heterocyclic structures as backbones	Many FDA-approved drugs are based on heterocyclic structures	Heterocycles form the core of therapeutic drugs across various fields
9	Continued exploration of heterocycles	Ongoing research in heterocycles is crucial for new drug discovery	Necessary for addressing unmet medical needs and expanding drug therapies
10	Impact on global healthcare	Heterocyclic-based drugs have transformed global healthcare	Played a major role in the advancement of treatments across therapeutic areas

**Figure:**
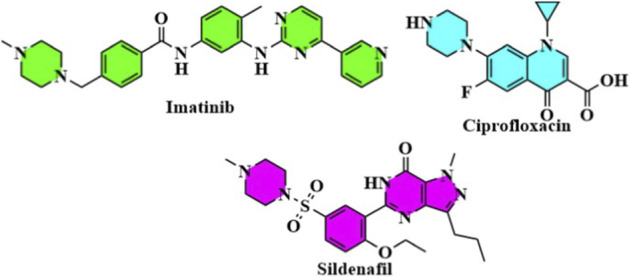


## 3 Strategic applications in pharmaceutical industries

### 3.1 Drug discovery and development strategies involving heterocycles

Unique chemical and biological structures of heterocyclic compounds as drugs have inspired innovative strategies that help to usher in the discovery and development of heterocyclic compounds as drugs. In the initial stages of discovery, a high-throughput screening (HTS) platform is employed to identify target heterocyclic compounds exhibiting high biological activity. Researchers are screening large libraries of compounds to find lead candidates that bind well to biomolecular targets relevant to a disease, and many of these compounds have heterocycles. Once a lead hits the table SAR studies is employed to optimize the potency, selectivity and physicochemical properties of the molecule.

Enabling the techniques for Heterocyclic compounds were easily built up, as simple derivatives of the Heterocyclic ring can induce a large difference in the biological activity. It is known that changing a pyridine or quinoline ring to another structure can improve the binding affinity for a correlative receptor/enzyme. Medicinal chemistry techniques such as combinatorial chemistry and rational drug design are also utilized to develop new heterocyclic derivatives that demonstrate improved efficiency and safety profiles. Tools for computational modeling such as molecular docking and molecular dynamics simulations may also help predict how the heterocycles will interact with their targets, which can help inform the optimization process. Finally, heterocyclic based drugs must pass through preclinical and clinical trials to prove their safety, efficacy and regulation compliance. Heterocyclic compounds are still central to the development of novel therapeutic agents in numerous therapy areas because they have excellent medicinal properties that are well known (Kumar and Goel, 2022; Rizzo et al., 2023).

### 3.2 Design and synthesis of heterocyclic derivatives for therapeutic uses

**FIGURE 3:**
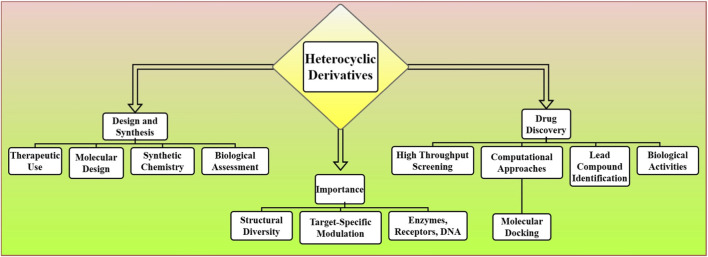
The process of designing and synthesizing heterocyclic derivatives for therapeutic use, emphasizing molecular design, synthetic chemistry, biological assessment, and high throughput screening in drug discovery.

The design and synthesis of heterocyclic derivatives for therapeutic use, and a systematic approach to molecular design, synthetic chemistry, and biological assessment in drug discovery. Compounds containing heterocycles are arguably the most important class in drug discovery as they possess structural diversity and many of the biologically relevant targets ranging from enzymes to receptors to DNA can be modulated in a target-specific manner from interacting with them. Basically, it begins with high throughput screening or computational approaches (for example, molecular docking), which usually lead to the identification of heterocycles as lead compounds with possible biological activities Figure 3. By altering the heterocyclic configuration including the heteroatoms (nitrogen, oxygen, sulfur) or ring size researchers can improve potency, selectivity, and pharmacokinetic properties. Report synthetic strategies that involve cyclization reactions, metal-catalysed processes and multicomponent reactions to produce the heterocyclic rings. After synthesis, the compounds are tested *in vitro* and *in vivo* for their potency, toxicity, and pharmacokinetic properties. Numerous other heterocyclic derivatives have also been demonstrated to have powerful therapeutic action against a large variety of diseases such as cancer, infections and neurological disorders, making heterocycles a highly valuable class of compounds in medicinal chemistry. And their constant improvement is driving innovation in drug development (Bhutani et al., 2021).

### 3.3 Natural products background in drug discovery and discovered natural products in market

Heterocycles are essential in developing drugs like azithromycin (antibiotic), oseltamivir (antiviral), and imatinib (anticancer). Their structural flexibility allows for the optimization of drug-receptor interactions (Kumar and Goel, 2022; dos Santos et al., 2021).

Natural product chemistry has led to the discovery of a number of blockbuster heterocyclic drugs. Quinine (antimalarial), Camptothecin (anticancer parent of irinotecan and topotecan) and the beta-lactam antibiotics (penicillin scaffold) are all examples of naturally occurring heterocycles which have received extensive commercial use. Such natural product-inspired or natural product-derived heterocycles remain dominant in the therapeutic pipelines in the modern pharmaceutical environment. Newman and Cragg (2020) (Newman and Cragg, 2020) state that more than 50% of all drugs approved by FDA are natural products or derivatives, and a large percentage of those, have heterocyclic cores. This highlights why it is important to merge natural product chemistry and synthetic approaches to heterocyclic drugs development. Paclitaxel (Taxol) analogs, macrolide antibiotics (azithromycin), and alkaloid-based anticancer agents are examples of markets which have brought in millions of dollars in revenue across the pharmaceutical industry around the globe.

### 3.4 Method development of producing drugs in market

When a heterocyclic compound is to be transferred to the commercial environment, the development of the methods should be thoroughly conducted. This entails process chemistry optimization in order to enhance yield, minimize impurities and scale synthesis effectively. Catalytic hydrogenation, regioselective functionalization and continuous flow chemistry is consistently used in industrial synthesis of heterocyclic drugs to provide high throughput. Indicatively, the production of Imatinib (Gleevec) by commercial synthesis utilized a convergent multi-step synthesis that has been optimized to utilize efficient heterocyclic coupling techniques in large-scale production (kilograms-scale). On the same note, wasting of proton pump inhibitors (e.g. Omeprazole, Lansoprazole) is based on scalable benzimidazole synthesis protocols. These method development undertakings are part and parcel of regulatory submission, because both the FDA and the ICH guidelines specify that manufacturing processes should be both validated and reproducible. The shift to continuous manufacturing and process analytical technology (PAT) has also increased the efficiency of heterocyclic drugs at lower cost and shorter time to market (Khan et al., 2022).

### 3.5 Green synthesis and multicomponent reaction (MCR) approach to drug discovery

The approaches of green chemistry and multicomponent reactions (MCRs) are radically different paradigms in heterocyclic synthesis, and provide operationally simple, atom-economical, and sustainable routes to complex molecular structures. MCRs allow creating several bonds at once during the process, enhancing the synthetic efficiency numerous times and decreasing the level of waste. It is worth noting that PEG-200-assisted one-pot three-component synthesis of functionalized N-amino-3-cyano-2-pyridones without volatile catalysts indicates how benign green solvents can be used to promote the formation of heterocyclic rings (Das et al., 2023). Likewise, the preparation of 5-membered ring fused pyrimidine derivatives using anisocyanide-based one-pot three-component reactions and 6-membered thiazine-dicarboxylates using MCR strategies is similarly illustrative of the versatility of this strategy (Mohlala et al., 2020; Mohlala RL et al., 2021). MCRs have demonstrated an impressive development during the last 5 years and turned out to be an effective and easily accessible alternative to the classical multi-step synthetic pathways, as emphasize (Mohlala et al., 2024). The implementation of MCR and green chemistry principles decreases the amount of solvents used, limits the number of dangerous products, and decreases the amount of carbon footprint of pharmaceutical production, which is very consistent with the overall objectives of sustainability across the globe and the values promoted by the ACS Green Chemistry Institute (Zhang et al., 2020).

### 3.6 Atom economy and waste generated by pharmaceutical companies

A classical methodology of measuring green chemistry is atom economy which is a metric proposed by Zhang et al. (2020) to indicate the efficiency of a chemical reaction by determining the proportion of reactant atoms that are incorporated into the desired product. Conventional multi-step chain-reaction heterocyclic reactions have low atom economy with large yields of byproducts and solvent waste. The E-factor of the pharmaceutical industry is one of the highest of the chemical manufacturing industries, with the estimates of E-factor of active pharmaceutical ingredient (API) synthesis ranging at 25–100 kg waste/kg product (Federsel, 2022). This has seen the leading pharmaceutical firms, such as Pfizer, AstraZeneca, and GSK, embrace the idea of green chemistry and monitor such measures as Process Mass Intensity (PMI), Carbon Efficiency and Reaction Mass Efficiency (RME). Implementation of MCRs, catalytic procedures, solvent recovery technologies, and downsizing procedures have made possible quantifiable waste production decreases. Additional advances in atom economy in the production of heterocyclic drugs will come with future integration of biocatalysis, electrochemical synthesis and continuous flow reactors.

## 4 Business perspectives on heterocyclic compounds

### 4.1 Market trends and economic impact of heterocyclic drugs

**FIGURE 4:**
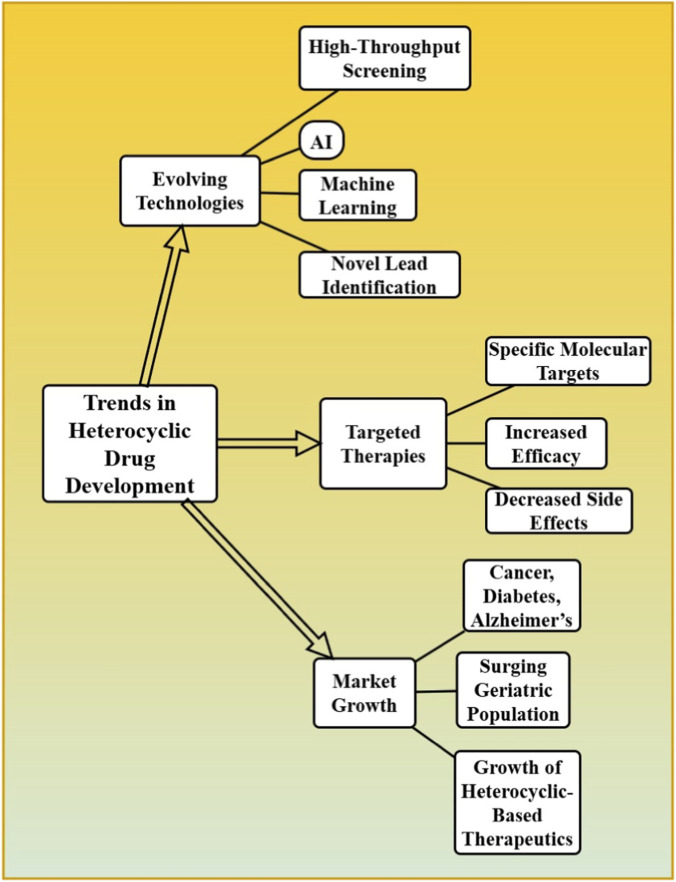
The trends in heterocyclic drug development, highlighting evolving technologies like AI and high-throughput screening, targeted therapies for increased efficacy, and the growing market driven by disease prevalence.

The heterocyclic drugs are a class that accounts for a significant portion of the pharmaceutical market, as their structure provides versatile applications in various therapeutic areas. Many human health applications rely on these compounds, including chronic disease, cancer, infectious diseases, and neurological disorders, which contribute to the global healthcare market. The heterocyclic-based drugs market is booming nowadays with the driven from traditional drug classes as well as growing needs for new therapies in recent years. The trends have led to an increase in the development of heterocyclic drugs as drug discovery technologies keep evolving i.e. high-throughput screening, artificial intelligence (AI), and machine learning for novel lead identification (dos Santos et al., 2021; Sharma et al., 2020). Furthermore, the use of targeted therapies is becoming more popular in this way heterocyclic compounds are used to talk with specific molecular targets which increases the efficacy of the drug as well as decreases side effects. The increasing incidence of diseases like cancer, diabetes and Alzheimer’s along with surging geriatric population across the globe is augmenting growth of heterocyclic based therapeutics Figure 4. In 2022, the worldwide pharmaceutical market stood at about USD 1.48 trillion and will have grown to USD 2.3 trillion by 2030 (CAGR: 5.8%; IQVIA, 2023) (Huang-Lin et al., 2026). It is estimated that a small molecule drug approved by the FDA is made up of heterocyclic compounds 70-85 percent. Certain market statistics: (i) Imatinib (Gleevec) earned the highest sales worldwide amounted to USD 4.7 billion annually prior to the generic penetration. (ii) USD 65 billion globally was generated by the class of kinase inhibitor, which is mostly heterocyclic, in 2022. (iii) USD 4.5 billion market Proton pump inhibitors (benzimidazole class). (iv) The Antiretroviral drugs (heterocyclic NNRTIs and NRTIs) provided more than USD 30 billion in 2022. R&D cost data: To introduce a new heterocyclic drug to the market, the average cost is estimated at USD 1.3–2.6 billion, and the mean time spent on the development of a new drug is 10–15 years. Market share by country: United States (almost 45 percent), Europe (almost 25 percent), Japan (almost 7 percent), emerging markets, such as China and India (almost 15 percent).

As heterocyclic drugs are an important economical part of medications. It is estimated that heterocyclic‐based drugs account for the sale of multi‐billion dollar product from worldwide pharmaceutical industries. R&D is responsible for billions in annual sales of drugs, including compounds like Imatinib a heterocyclic anticancer therapeutic. As a result, this creates the basis for job creation as developing, manufacturing and commercially selling these drugs will need the effort of a multitude of industries from biotechnology to pharma and health services. The application of these methodologies in synthesising therapeutics not only helps in increasing the throughput of drug discovery but also lead to development of more efficient therapeutics that lead to overall reduction in healthcare costs considering them being potentially applicable in treatment of chronic diseases where long-term care is involved.

### 4.2 Investment opportunities in heterocyclic chemistry

**FIGURE 5:**
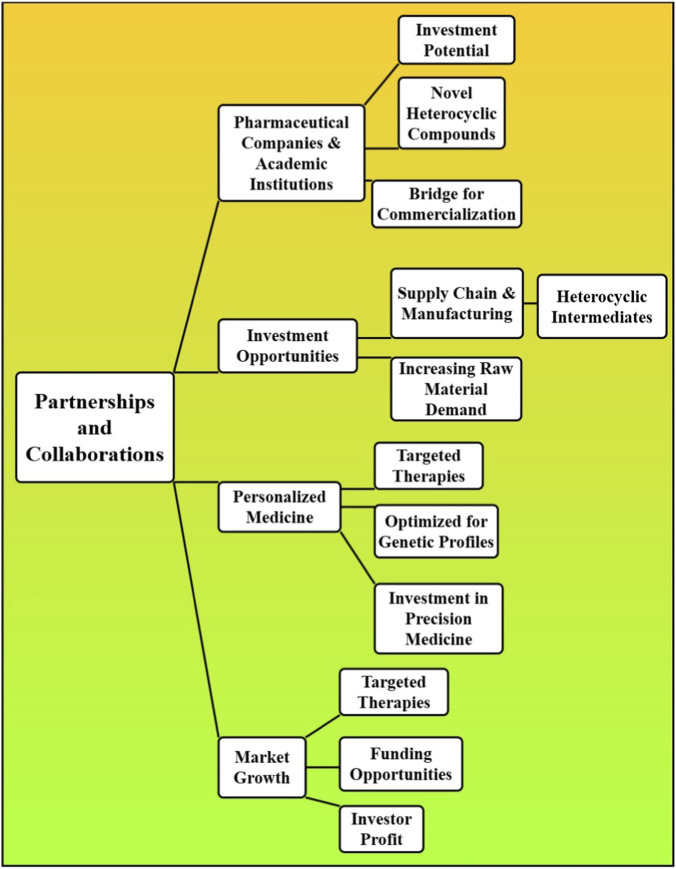
The partnerships and investment opportunities in heterocyclic drug development, emphasizing collaborations between academia and industry, manufacturing of intermediates, and the growth of personalized and targeted therapies.

Collectively, heterocyclic chemistry is a major area of investment across the pharmaceutical and biotechnology sectors, with increasing prospects for value generation both through novel drug therapies and pharmaceutical methods achieving key parameters beneficial to the treatment of complex morbidities. As the healthcare industry progresses, heterocyclic compounds remain a cornerstone of drug discovery with high-value opportunities for both investors and pharmaceutical companies. Investment in heterocyclic chemistry is on the increase as pharmaceutical companies invest into Research and Development (R&D) of new heterocyclic compounds to address cancer, infectious diseases (including antibiotic-resistant organisms), neurological conditions and other unmet clinical needs. The emergence of computational chemistry technology, including machine learning and artificial intelligence for drug design, is fast-tracking the search for new heterocyclic leads, paving the way for innovative therapies and improved patient outcomes. Investors have the potential to find aloe-suitable opportunities in biotech start-ups and research-and-development-focused companies that the next-generation of heterocycles-based cures (Dai et al., 2018; Pfab et al., 2021). The partnerships and collaborations between pharmaceutical companies and academic institutions also have high examples of investment potential. As a result, many universities and research centers design novel heterocyclic compounds, and agreements among these institutes and commercial partners form a bridge to facilitate further commercialization. Investment opportunities also lie within the companies focusing on the supply chain and manufacturing of heterocyclic intermediates as the demand for these raw materials increase. As personalized medicine continues to gain prominence, the demand for targeted therapies increases, providing further investment potential with heterocyclic compounds that can be optimized for individual genetic profiles or molecular pathways Figure 5. With the growth of precision medicine, the heterocyclic drugs’ market and investors could profit by funding companies creating targeted therapies. The increasing necessity for newly developed effective therapies within the pharmaceutical industry is an excellent reason to consider investing in heterocyclic chemistry due to the whole creative process involving drug discovery, synthesis, and manufacturing. International pharma R&D investing statistics: The pharmaceutical sector is estimated in 2022 to have invested USD 244 billion in R&D across the world (IFPMA, 2023) (Palache et al., 2025). Investments in heterocyclic drug programs by Biotech: USD 18.3 billion venture capital in 2021. Cases of specific investment: USD 6 billion investment 2020–2023 AstraZeneca oncology pipeline (80% heterocyclic). Partnership case: Pfizer-BioNTech COVID collaboration earned USD 37 billion revenue in 2021, with the help of heterocyclic antiviral platforms. Amended 4.3 contains: Average US patent filing cost: USD 15,000-30,000 (basic); USD 50,000-150,000 (international PCT). Repayment of patent fees: USD 800-7,400/annually (US PTO). Market exclusivity 20-year patent protection + Hatch-Waxman extension of 5 years.

### 4.3 Intellectual property and patent landscape

Heterocyclic chemistry represents a considerable domain of intellectual property (IP) and patents due to its importance in the development of pharmaceuticals, agrochemicals and materials science. Heterocyclic compounds play key role in the pharmaceutical industry as they are components of diverse drugs with specific biological activities due to their various structures and functional groups. The growing need for new therapeutic agents such as anticancer, antiviral and antimicrobial agents has led to a growing of research and patent applications of heterocyclic compounds. The patents granted for novel heterocyclic molecules or the methods of synthesis give the exclusive rights for a limited period, allowing companies to recover the investments incurred in research and development. Additionally, exclusivity in the market is further guaranteed by the protection of manufacturing processes and formulations (Kabir and Uzzaman, 2022; Marshall et al., 2024; Mathew et al., 2023). Developing trends in heterocyclic chemistry patenting Abstract: Here, we investigate the patent landscape in heterocyclic chemistry, observing a more and more dynamic patent landscape is emerging with increasing focus on combinatorial chemistry and multi-target compounds. Such patenting goes beyond just individual compounds and extends to the processes, intermediates, and polymorphs that impact a molecule’s efficacy; A practice known as strategic patenting. Licensing agreements, collaboration, and cross-licensing have become basic parts of the IP methodology, which enable organizations to build their portfolio and settle into new markets. As new compounds and drug candidates emerge, and improvements are made in synthetic methods, the patent landscape around heterocyclic chemistry remains dynamic, with numerous opportunities for innovation and intellectual property protection, especially in the field of drug discovery and materials technology.

## 5 Stringent regulatory requirements

**FIGURE 6:**
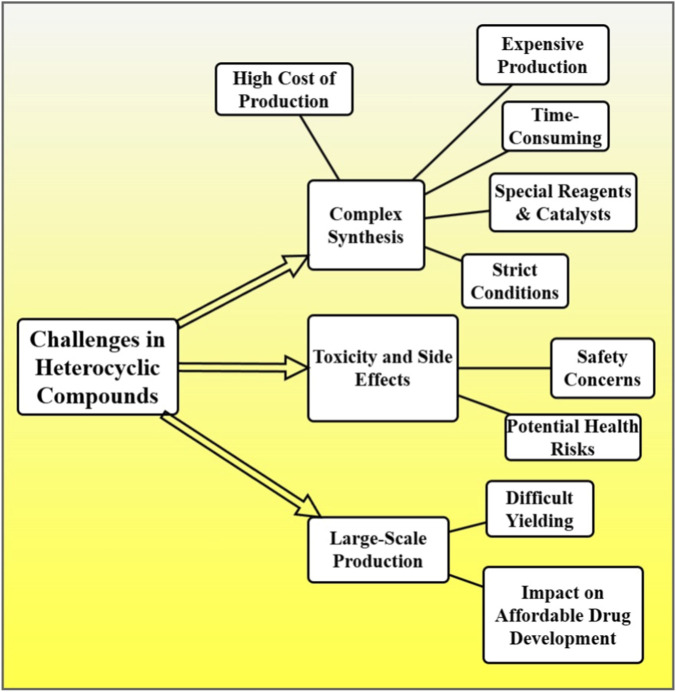
The challenges in the commercial and therapeutic efficacy of heterocyclic compounds, focusing on complex synthesis, high production costs, toxicity concerns, and difficulties in large-scale production.

Various challenges and constraints restrict the commercial and therapeutic efficacy of heterocyclic compounds as pharmaceuticals intermediate. One of the greater obstacles is their complex synthesis. Many heterocyclic heterocycles need to be subjected to complicated synthetic pathways which can be expensive to produce and take time and yield large-scale production. This is the requirement of special reagents, catalysts, and strict conditions; thus, the increased cost of production potentially poses a significant challenge to cheap drug development. Another concern is toxicity and side effects (Kumar and Goel, 2022; Qadir et al., 2022). The biological activities of heterocyclic compounds have been extensively studied, but not always the safety profile is predictable. This complicates clinical development of such compounds, which could be cytotoxic or, do so in a way that elicits adverse reactions. To ensure these molecules work as well as are safe you need rigorous, and expensive, and time consuming, testing Figure 6.

Because of stringent regulatory requirements, drug discovery for heterocyclic compounds is taxing, causing delays to the approval process. The demand for robust preclinical and clinical data to prove efficacy as well as safety may lead to long timelines and substantial costs. The patent protection is crucial to prevent unauthorized use of new heterocyclic compounds, obtaining a patent can be a daunting task potential patentability, patent exclusivity over time, the expiration of patents, etc. In fact, given competition in the marketplace, particularly from generic manufacturers upon expiration of patents, the long-term profitability for heterocyclic-based drugs is likewise very limited (Bamakan et al., 2021; Mohtashami et al., 2020; Pai et al., 2022). These undertakings open new avenues for crossing hurdles faced by heterocyclic compounds in terms of their pharmaceutical advances, strategic partnerships, technology advancements, and holding an effective business model.

(1) FDA Requirements: IND filing, based on Phase I-III clinical trial data; NDA filing, based on full CMC (Chemistry, Manufacturing, and Controls) data; PDUFA review, 10–12 months (standard), 6 months (priority review). Acceptance threshold of heterocyclic API purity is defined by ICH Q6A guidelines, and genotoxic impurity limits of (ICH M7) are of particular interest to heterocyclic nitrogen-containing systems, which form mutagenic impurities. (2) EMA Requirements: CHMP determination of benefit-risk ratio; EMA REACH compliance of heterocyclic synthesis of chemical intermediates. (3) Particular to the Heterocyclic Drugs: N-oxide formation, ring-opening during physiological conditions and photodegradation are typical stability issues that demand photostability testing according to the ICH Q1B. Testing of impurities of nitrosamines (FDA guidance 2020–2023) (Nugent et al., 2025) has now been required in a wide range of heterocyclic drugs. (4) Patent-Regulatory Intersection: United States Hatch-Waxman Act allows a 30-month stay period on patent challenge by generic firms (heterocyclic drug, a key business strategy) which is a vital market exclusivity on top of the underlying basic patent term.

## 6 Future directions and innovation opportunities

**FIGURE 7:**
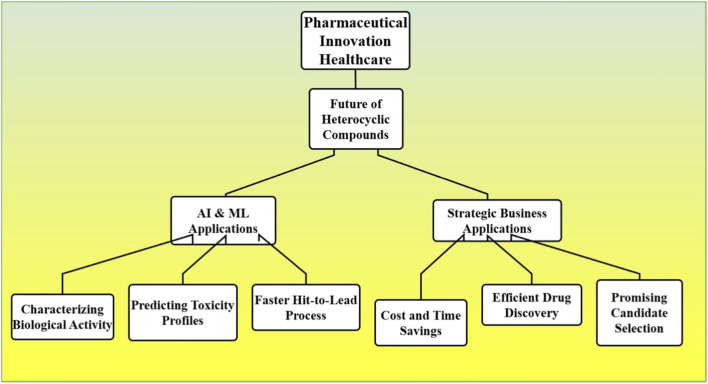
The future of heterocyclic compounds in pharmaceutical innovation, emphasizing the role of AI and machine learning in characterizing biological activity, predicting toxicity, and accelerating the drug discovery process.

The first crucial direction is the implementation of artificial intelligence (AI) and machine learning (ML) in the drug bionic process. Artificial intelligence (AI) based algorithms can quickly characterize the biological activity and toxicity profiles of newly synthesized heterocyclic compounds facilitating the selection of promising candidates for development (Kabir and Uzzaman, 2022). By saving time and the costs involved with discovering drugs, this technology can allow for the hit-to-lead process to occur significantly faster Figure 7.

The challenge provides an opportunity for businesses to explore and develop greener synthesis routes within the pharmaceutical industry as a response to increasing global demand for sustainable and eco-friendly manufacturing processes. Furthermore, greening chemistry not only minimizes the footprint on nature, but it also increases cost-efficiency and ensures that business will be sustainable for long into the future (Qadir et al., 2022; Wang et al., 2020).

Personalized medicine is another field that can use a helping hand and heterocyclics have all the prospects to progress in this area. Genomics and biomarker discovery have enabled drugs to target more specific pathways and heterocycles can be designed to target specific genetic mutations or disease pathways. This opens door to potential niche, high-value therapies, particularly in segments such as oncology and rare diseases. Further, the combination therapy of heterocyclic compounds is an interesting topic to study on. Multidrug compounds have been traditionally important in pharmacology and recognizing compound features by combined multidrug action with other drug classes or poly-pharmacology approach can overcome drug resistance and therapeutic efficiency in malignant diseases as cancer and infectious diseases. There is potential for big opportunities and game-changing step towards heterocyclic therapeutics by working with those innovations and utilizing sustainable instrumental practices in pharmaceutical industry (Gönül et al., 2023; Parthiban, 2023; Wang, 2023).

## 7 Discussion

The strategic application of heterocyclics in pharmaceutical development presents both remarkable opportunities and challenges. Their structural aspects have inspired discovery of such compounds for decades, which allows designing of molecules with diverse biological activities. From anticancer specifically to antimicrobial agents to agents acting against neurodegenerative diseases, heterocyclic compounds give potent drugs to become the new remedy to fill the gap of unmet medical needs. Synthesis is typically a multi-step process and scaling it up is costly, rendering the process cost prohibitive (Arabmarkadeh et al., 2021; Gadiya et al., 2023; Heravi and Zadsirjan, 2020). Moreover, the toxicity and safety profile of heterocyclic compounds may be difficult to predict, resulting in drugs that are difficult to bring to market. Add to this regulatory hurdles, huge testing efforts and compliance with international standards are required before market authorization. But personalized medicine, the use of artificial intelligence (AI) and machine learning in drug discovery, and green chemistry are some new opportunities that can potentially help overcome these challenges. Pharmaceutical companies can also leverage efforts of introducing new heterocyclic scaffolds for drug discovery with other companies that are completing data through strategic partnerships and licensing agreements or keeping the production sustainable from multiple sources.

## 8 Conclusion

We believe heterocyclic compounds can drive pharmaceutical innovation against traditional and rare disease, and can be an important value driver for the pharma industry. While these factors create challenges for synthesis, safety, and regulatory approval, the continued development and advances in fields such as AI and machine learning, personalized medicine, and green chemistry can also ameliorate these concerns. Moreover, businesses will seek to improve their competitiveness with the aid of joint ventures and licensing agreements. Concisely, heterocyclic substances hold key components in drug innovation, establishing an appealing territory for corporate exploration.
